# Diaqua­bis(5-fluoro-2-hydroxy­benzoato-κ*O* ^1^)zinc(II)

**DOI:** 10.1107/S1600536809005716

**Published:** 2009-02-28

**Authors:** Diana Rishmawi, Jennifer Kelley, Mark D. Smith, LeRoy Peterson, Hans-Conrad zur Loye

**Affiliations:** a Chemistry Department, Francis Marion University, Florence, South Carolina 29501, USA; b Department of Chemistry and Biochemistry, University of South Carolina, Columbia, South Carolina 29208, USA

## Abstract

The title compound, [Zn(C_7_H_4_FO_3_)_2_(H_2_O)_2_], is a monomeric Zn^II^ complex. The Zn^II^ atom, which lies on a twofold rotation axis, is situated in a distorted tetra­hedral environment composed of two monodentate carboxlyate O atoms and two water O atoms. O—H⋯O hydrogen bonds link these units, forming sheets that are stacked along the *c* axis.

## Related literature

For general background, see: Ellsworth & zur Loye (2008 ▶); Janiak (2003 ▶); Mehrotra & Bohra (1983 ▶); Wasuke *et al.* (2005 ▶). For related structures, see: Brownless *et al.* (1999 ▶); Wang *et al.* (2006 ▶).
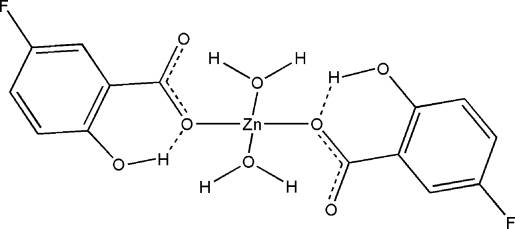


## Experimental

### 

#### Crystal data

[Zn(C_7_H_4_FO_3_)_2_(H_2_O)_2_]
*M* *_r_* = 411.61
Monoclinic, 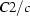

*a* = 15.3096 (10) Å
*b* = 5.4706 (4) Å
*c* = 17.7741 (12) Å
β = 91.674 (1)°
*V* = 1487.99 (18) Å^3^
*Z* = 4
Mo *K*α radiation
μ = 1.72 mm^−1^
*T* = 150 K
0.16 × 0.12 × 0.05 mm

#### Data collection

Bruker SMART APEX CCD diffractometer
Absorption correction: multi-scan (*SADABS*; Bruker, 2001 ▶) *T*_min_ = 0.893, *T*_max_ = 1.000 (expected range = 0.820–0.918)
8435 measured reflections
1520 independent reflections
1341 reflections with *I* > 2σ(*I*)
*R*_int_ = 0.053

#### Refinement

*R*[*F* ^2^ > 2σ(*F* ^2^)] = 0.035
*wR*(*F* ^2^) = 0.081
*S* = 1.09
1520 reflections
126 parameters
3 restraints
H atoms treated by a mixture of independent and constrained refinement
Δρ_max_ = 0.43 e Å^−3^
Δρ_min_ = −0.27 e Å^−3^

### 

Data collection: *SMART* (Bruker, 2007 ▶); cell refinement: *SAINT-Plus* (Bruker, 2007 ▶); data reduction: *SAINT-Plus*; program(s) used to solve structure: *SHELXS97* (Sheldrick, 2008 ▶); program(s) used to refine structure: *SHELXL97* (Sheldrick, 2008 ▶); molecular graphics: *DIAMOND* (Brandenburg, 1999 ▶); software used to prepare material for publication: *SHELXTL* (Sheldrick, 2008 ▶).

## Supplementary Material

Crystal structure: contains datablocks global, I. DOI: 10.1107/S1600536809005716/hy2183sup1.cif

Structure factors: contains datablocks I. DOI: 10.1107/S1600536809005716/hy2183Isup2.hkl

Additional supplementary materials:  crystallographic information; 3D view; checkCIF report

## Figures and Tables

**Table:** 

Zn1—O4	1.966 (2)
Zn1—O1	1.9716 (17)

**Table:** 

O4—Zn1—O4^i^	100.61 (13)
O4—Zn1—O1	121.01 (8)
O4—Zn1—O1^i^	94.50 (8)
O1—Zn1—O1^i^	124.62 (11)

Symmetry code: (i) 
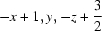
.

**Table 2:** Hydrogen-bond geometry (Å, °)

*D*—H⋯*A*	*D*—H	H⋯*A*	*D*⋯*A*	*D*—H⋯*A*
O3—H3⋯O1	0.803 (18)	1.84 (2)	2.564 (3)	149 (3)
O4—H4*A*⋯O2^ii^	0.834 (18)	1.83 (2)	2.641 (3)	162 (3)
O4—H4*B*⋯O3^iii^	0.834 (19)	1.89 (2)	2.711 (3)	170 (4)

Symmetry codes: (ii) 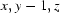; (iii) 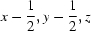.

**Fig. 1.**
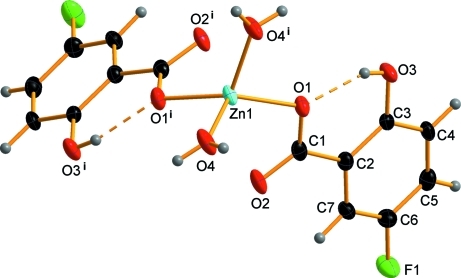
Molecular structure of the title compound. Displacement ellipsoids are drawn at the 50% probability level. Hydrogen bonds are represented by dashed lines. [Symmetry code: (i) -x+1, y, -z+3/2.]

**Fig. 2.**
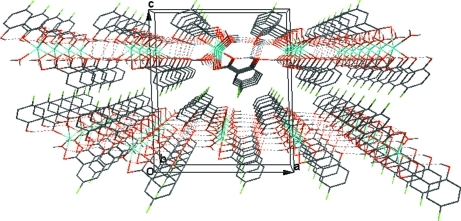
View of the crystal packing in the title compound. All H atoms except for those of water and the hydroxyl group are omitted for clarity. Hydrogen bonds are represented by dashed lines.

## supplementary crystallographic information

### Comment

Metal carboxylate complexes have long been an extensively studied class of compounds (Mehrotra & Bohra, 1983), and in recent years they have become a major focus of study due to their potentally useful properties (Janiak, 2003; Wasuke *et al.*,  2005). As a continuation of our own studies (Ellsworth & zur Loye, 2008), we report here the crystal structure of the title compound.

The structure of the title compound is built from the monomeric complex of formula Zn(5-fsalcyl)_2_(H_2_O)_2_ (Fig. 1) (5-fsalcyl = 5-fluorosalicylate). The asymmetric unit consists of one Zn^II^ atom that lies on a twofold rotation axis, one 5-fsalcyl ligand, and one water molecule. The coordination environment of the Zn^II^ atom is that of a distorted tetrahedron consisting of two equivalent O atoms from two monodentate carboxylates, and two equivalent O atoms from two water molecules. All four Zn—O bond distances fall within the normal range, with an average length of 1.969 (2) Å. It is worth noting that for the carboxylate O2 atom, the Zn···O2 distance of 2.692 (2)Å falls outside the range considered normal for a Zn—O coordination bond (Wang *et al.*, 2006).

Due to its monodentate binding mode, the 5-fsalcyl carboxylate group adopts a highly asymmetrical configuration. This is manifested in a C1—O1 distance [1.289 (3) Å] for the coordinating O atom that is noticeably longer than the C1—O2 distance [1.246 (3) Å] corresponding to the noncoordinating O atom. In addition, the carboxylate group of the 5-fsalcyl ligand is twisted with a dihedral angle of 9.7 (2) ° with respect to the phenyl ring. As is typical for salicylates, the hydroxyl group of 5-fsalcyl is internally hydrogen bonded to its carboxylate O1 that is located on the same side of the ligand (Brownless *et al.*, 1999).

The monomeric units are hydrogen bonded into chains that are themselves hydrogen bonded into sheets that are stacked along the *c* axis (Fig. 2).

### Experimental

All chemicals and solvents were purchased from commercial sources and used without further purification. 5-Fluorosalicylic acid (3 mmol) was added to 100 ml of water and subsequently brought to pH 6.5 by the addition of 3*M* NaOH with constant stirring. To this solution was added 10 ml of a 0.10 *M* solution of Zn(NO_3_)_2_.6H_2_O. Single crystals of the title compound were formed in four weeks after complete evaporation of the solution under ambient conditions.

### Refinement

H atoms bonded to C atoms were positioned geometrically and refined as riding atoms. O-bound H atoms were located in a difference Fourier map and refined isotropically, with their O—H distances restrained to 0.84 (2) Å.

### Crystal data

**Table:**

[Zn(C_7_H_4_FO_3_)_2_(H_2_O)_2_]	*F*(000) = 832
*M**_r_* = 411.61	*D*_x_ = 1.837 Mg m^−^^3^
Monoclinic, *C*2/*c*	Mo *K*α radiation, λ = 0.71073 Å
Hall symbol: -C 2yc	Cell parameters from 2066 reflections
*a* = 15.3096 (10) Å	θ = 2.7–24.1°
*b* = 5.4706 (4) Å	µ = 1.72 mm^−^^1^
*c* = 17.7741 (12) Å	*T* = 150 K
β = 91.674 (1)°	Plate, colorless
*V* = 1487.99 (18) Å^3^	0.16 × 0.12 × 0.05 mm
*Z* = 4	

### Data collection

**Table:**

Bruker SMART APEX CCD diffractometer	1520 independent reflections
Radiation source: fine-focus sealed tube	1341 reflections with *I* > 2σ(*I*)
graphite	*R*_int_ = 0.053
φ and ω scans	θ_max_ = 26.4°, θ_min_ = 2.3°
Absorption correction: multi-scan (*SADABS*; Bruker, 2001)	*h* = −18→18
*T*_min_ = 0.893, *T*_max_ = 1.000	*k* = −6→6
8435 measured reflections	*l* = −22→22

### Refinement

**Table:**

Refinement on *F*^2^	Primary atom site location: structure-invariant direct methods
Least-squares matrix: full	Secondary atom site location: difference Fourier map
*R*[*F*^2^ > 2σ(*F*^2^)] = 0.035	Hydrogen site location: mixed
*wR*(*F*^2^) = 0.081	H atoms treated by a mixture of independent and constrained refinement
*S* = 1.09	*w* = 1/[σ^2^(*F*_o_^2^) + (0.0397*P*)^2^ + 0.7409*P*] where *P* = (*F*_o_^2^ + 2*F*_c_^2^)/3
1520 reflections	(Δ/σ)_max_ < 0.001
126 parameters	Δρ_max_ = 0.43 e Å^−^^3^
3 restraints	Δρ_min_ = −0.27 e Å^−^^3^

### Fractional atomic coordinates and isotropic or equivalent isotropic displacement parameters (Å^2^)

**Table:**

	*x*	*y*	*z*	*U*_iso_*/*U*_eq_	
Zn1	0.5000	0.08063 (8)	0.7500	0.01954 (15)	
C1	0.56321 (16)	0.4151 (5)	0.65619 (14)	0.0204 (5)	
C2	0.62447 (16)	0.5945 (5)	0.62352 (14)	0.0199 (5)	
C3	0.71316 (16)	0.6031 (5)	0.64632 (14)	0.0191 (5)	
C4	0.76803 (16)	0.7820 (5)	0.61874 (14)	0.0221 (6)	
H4	0.8278	0.7864	0.6346	0.026*	
C5	0.73553 (18)	0.9533 (5)	0.56829 (15)	0.0246 (6)	
H5	0.7724	1.0775	0.5495	0.029*	
C6	0.64862 (18)	0.9416 (5)	0.54547 (15)	0.0248 (6)	
C7	0.59321 (16)	0.7666 (5)	0.57150 (14)	0.0223 (6)	
H7	0.5339	0.7624	0.5543	0.027*	
O1	0.59620 (11)	0.2481 (3)	0.69963 (10)	0.0233 (4)	
O2	0.48283 (11)	0.4310 (3)	0.64435 (11)	0.0265 (4)	
F1	0.61747 (11)	1.1088 (3)	0.49488 (10)	0.0391 (5)	
O3	0.74885 (12)	0.4400 (4)	0.69676 (11)	0.0266 (4)	
H3	0.7120 (18)	0.341 (5)	0.7060 (18)	0.044 (10)*	
O4	0.42296 (13)	−0.1489 (4)	0.69475 (13)	0.0305 (5)	
H4A	0.437 (2)	−0.271 (5)	0.6700 (16)	0.039 (10)*	
H4B	0.3689 (13)	−0.132 (8)	0.691 (2)	0.068 (14)*	

### Atomic displacement parameters (Å^2^)

**Table:**

	*U*^11^	*U*^22^	*U*^33^	*U*^12^	*U*^13^	*U*^23^
Zn1	0.0113 (2)	0.0184 (2)	0.0290 (3)	0.000	0.00249 (16)	0.000
C1	0.0169 (13)	0.0199 (13)	0.0245 (14)	0.0015 (10)	0.0036 (10)	−0.0063 (11)
C2	0.0174 (12)	0.0209 (13)	0.0214 (13)	−0.0025 (11)	0.0021 (10)	−0.0012 (11)
C3	0.0149 (12)	0.0239 (14)	0.0186 (13)	0.0015 (10)	0.0019 (10)	−0.0007 (11)
C4	0.0118 (12)	0.0293 (15)	0.0252 (14)	−0.0026 (11)	0.0016 (10)	−0.0009 (12)
C5	0.0224 (14)	0.0238 (15)	0.0277 (14)	−0.0068 (11)	0.0065 (11)	0.0003 (12)
C6	0.0233 (14)	0.0255 (15)	0.0256 (14)	0.0021 (12)	0.0000 (11)	0.0056 (12)
C7	0.0151 (13)	0.0263 (14)	0.0253 (14)	−0.0005 (11)	−0.0013 (10)	0.0008 (12)
O1	0.0150 (9)	0.0239 (10)	0.0312 (10)	0.0009 (8)	0.0054 (7)	0.0060 (8)
O2	0.0121 (9)	0.0233 (10)	0.0443 (12)	−0.0014 (8)	0.0025 (8)	−0.0025 (9)
F1	0.0282 (9)	0.0390 (10)	0.0500 (11)	0.0008 (8)	−0.0028 (8)	0.0232 (9)
O3	0.0132 (9)	0.0315 (11)	0.0350 (11)	−0.0020 (8)	−0.0020 (8)	0.0111 (9)
O4	0.0139 (10)	0.0277 (11)	0.0497 (13)	0.0020 (8)	−0.0017 (9)	−0.0155 (10)

### Geometric parameters (Å, °)

**Table:**

Zn1—O4	1.966 (2)	C4—C5	1.380 (4)
Zn1—O4^i^	1.966 (2)	C4—H4	0.9500
Zn1—O1	1.9716 (17)	C5—C6	1.381 (4)
Zn1—O1^i^	1.9717 (17)	C5—H5	0.9500
C1—O2	1.246 (3)	C6—F1	1.359 (3)
C1—O1	1.289 (3)	C6—C7	1.369 (4)
C1—C2	1.487 (4)	C7—H7	0.9500
C2—C7	1.394 (4)	O3—H3	0.803 (18)
C2—C3	1.406 (4)	O4—H4A	0.834 (18)
C3—O3	1.367 (3)	O4—H4B	0.834 (19)
C3—C4	1.388 (4)		
			
O4—Zn1—O4^i^	100.61 (13)	C5—C4—H4	120.1
O4—Zn1—O1	121.01 (8)	C3—C4—H4	120.1
O4^i^—Zn1—O1	94.50 (8)	C4—C5—C6	119.0 (2)
O4—Zn1—O1^i^	94.50 (8)	C4—C5—H5	120.5
O4^i^—Zn1—O1^i^	121.01 (8)	C6—C5—H5	120.5
O1—Zn1—O1^i^	124.62 (11)	F1—C6—C7	119.0 (2)
O2—C1—O1	121.2 (2)	F1—C6—C5	118.7 (2)
O2—C1—C2	121.3 (2)	C7—C6—C5	122.3 (2)
O1—C1—C2	117.4 (2)	C6—C7—C2	119.5 (2)
C7—C2—C3	118.6 (2)	C6—C7—H7	120.3
C7—C2—C1	119.8 (2)	C2—C7—H7	120.3
C3—C2—C1	121.6 (2)	C1—O1—Zn1	108.44 (15)
O3—C3—C4	117.2 (2)	C3—O3—H3	108 (2)
O3—C3—C2	122.1 (2)	Zn1—O4—H4A	128 (2)
C4—C3—C2	120.7 (2)	Zn1—O4—H4B	123 (3)
C5—C4—C3	119.9 (2)	H4A—O4—H4B	109 (4)
			
O2—C1—C2—C7	−7.8 (4)	C4—C5—C6—F1	−179.0 (2)
O1—C1—C2—C7	174.9 (2)	C4—C5—C6—C7	0.4 (4)
O2—C1—C2—C3	169.2 (2)	F1—C6—C7—C2	−179.9 (2)
O1—C1—C2—C3	−8.1 (4)	C5—C6—C7—C2	0.7 (4)
C7—C2—C3—O3	−179.6 (2)	C3—C2—C7—C6	−1.5 (4)
C1—C2—C3—O3	3.3 (4)	C1—C2—C7—C6	175.6 (2)
C7—C2—C3—C4	1.2 (4)	O2—C1—O1—Zn1	−8.5 (3)
C1—C2—C3—C4	−175.8 (2)	C2—C1—O1—Zn1	168.89 (17)
O3—C3—C4—C5	−179.3 (2)	O4—Zn1—O1—C1	73.68 (18)
C2—C3—C4—C5	−0.1 (4)	O4^i^—Zn1—O1—C1	178.91 (17)
C3—C4—C5—C6	−0.7 (4)	O1^i^—Zn1—O1—C1	−48.07 (15)

Symmetry codes: (i) −*x*+1, *y*, −*z*+3/2.

### Hydrogen-bond geometry (Å, °)

**Table:**

*D*—H···*A*	*D*—H	H···*A*	*D*···*A*	*D*—H···*A*
O3—H3···O1	0.80 (2)	1.84 (2)	2.564 (3)	149 (3)
O4—H4A···O2^ii^	0.83 (2)	1.83 (2)	2.641 (3)	162 (3)
O4—H4B···O3^iii^	0.83 (2)	1.89 (2)	2.711 (3)	170 (4)

Symmetry codes: (ii) *x*, *y*−1, *z*; (iii) *x*−1/2, *y*−1/2, *z*.
